# Sleep, sedentary behavior, and physical activity: A compositional data analysis of 24-hour movement behaviors and executive function in preschool children

**DOI:** 10.1371/journal.pone.0333563

**Published:** 2026-05-08

**Authors:** Ling Wang, Wanhong Luo, Yong Liu

**Affiliations:** 1 College of Sports Science, Changsha Normal University, Changsha, Hunan, PR China; 2 College of Physical Education, Hunan First Normal University, Changsha, Hunan, PR China; 3 College of Sports Science, Changsha Normal University, Changsha, Hunan, PR China; UBC: The University of British Columbia, CANADA

## Abstract

**Objective:**

This study aims to examine the relationship between 24-h movement behaviors and executive function in preschool children.

**Methods:**

This cross-sectional study included 266 preschool children (mean age: 3–6 years). Physical activity and sedentary behavior were measured using an accelerometer (ActiGraph GT3X-BT), sleep duration was assessed using sleep logs, and executive function was evaluated using the Early Years Toolbox. Compositional data analysis was then applied to examine the associations among these variables.

**Results:**

(1) The relative distribution of 24-h movement behaviors was significantly associated with inhibitory control, cognitive flexibility, and working memory (all *p* < 0.001), with a model explanatory power > 10%. The explanatory power was the highest for inhibitory control (16.3%). (2) After adjusting for other movement behaviors, sedentary behavior was negatively associated with inhibitory control [γ_1_^2^ = –0.11 (−0.17, −0.05), *p* < 0.001], cognitive flexibility [γ_1_^2^ = –1.45 (−2.29, −0.61), *p* = 0.001], and working memory [γ_1_^2^ = –0.58 (−0.97, −0.19), *p* = 0.004]. In contrast, sleep was positively associated with cognitive flexibility [γ_1_^1^ = 1.60 (0.26, 2.94), *p* = 0.020]. (3) When 15 min/day of sedentary behavior was isotemporally substituted with sleep, inhibitory control scores increased by 0.003 (0.0004, 0.006), and cognitive flexibility scores increased by 0.064 (0.021,0.106); conversely, replacing sleep with sedentary behavior resulted in a significant decline.

**Conclusion:**

Reallocating time from sedentary behavior to sleep was positively correlated with executive function, particularly cognitive flexibility. However, most other isotemporal substitution pathways did not show statistically significant effects, providing an important basis for future research to focus on specific behavioral reorganization strategies.

## 1. Introduction

Executive function (EF) is a foundational construct rooted in neuropsychology, first identified by observing patients who sustained frontal lobe injuries during wartime, manifesting pronounced impairments in self-control and behavioral execution, hence the coining of the term “EF” [1]. Despite extensive study, the academic community has not reached consensus on a uniform definition of EF. The concept is often distinguished along two dimensions: in its broad sense, EF encompasses the coordinated orchestration of multiple cognitive processes during information processing, whereas in its narrow sense, it typically refers to inhibitory control [2]. EF, or cognitive control, is a conscious, top-down neurocognitive mechanism that encompasses three core components: cognitive flexibility, inhibitory control, and working memory. These components facilitate goal-directed regulation of thoughts, actions, and emotions [3]. This tripartite framework has achieved widespread acceptance; therefore, this study operationalizes EF in three sub-functions: cognitive flexibility, inhibitory control, and working memory. EF, a high-level cognitive capability, develops rapidly during early childhood [4].

Extensive evidence has underscored the lasting impact of early childhood EF on various later-life outcomes. Early EF development correlates strongly with school readiness [5] and academic performance [6], as well as with adolescent behavioral patterns and broader adult outcomes, including health, socioeconomic status, and quality of life [7]. EF demonstrates considerable plasticity, especially during early childhood [8]. Emerging research points to 24-h movement behaviors (MBs), including physical activity (PA), sedentary behavior (SB),and sleep, as potentially critical influences on EF development [9,10]. Furthermore, MBs have been linked to diverse cognitive and psychological outcomes [11].

However, most extant studies have focused on the effects of individual behaviors in isolation, such as sleep [12], SB [13], and PA [14,15], without adequately addressing how these behaviors interact or compose across an entire day. This fragmented approach limits our understanding of how integrated daily patterns of MB influence EF. Accordingly, there is a growing recognition of the need for a holistic perspective that considers the combined effects of PA, SB, and sleep to inform more effective intervention strategies.

Recent studies using different methods have addressed this gap. McNeill et al. (2020) investigated the association between adherence to the 24-hour movement guidelines and both EF and psychosocial health in preschool children (mean age: 4.2 ± 0.6 years) [10]. Their findings regarding specific EF components were mixed. Specifically, while adherence was positively correlated with children’s cognitive flexibility and working memory in cross-sectional analyses, no significant longitudinal predictive effect of baseline adherence on these skills was observed. A Canadian study found that meeting both sleep and PA guidelines in early childhood (3–5 years) was related to overall developmental benefits and response inhibitory control, though SB showed no direct effect [16]. Lau et al. (2024) applied compositional and isotemporal reallocation analyses to assess the associations between MBs and EF in preschool children (3.8 ± 0.6 years), identifying significant links and demonstrating the practical value of reallocating time among behaviors for improving EF outcomes [17]. A more recent study further revealed that reallocating time from sleep or SB to moderate-to-vigorous PA (MVPA) can enhance cognitive flexibility in preschool children (3–5 years), reinforcing the importance of time-use intensity in EF development [18].

Nevertheless, existing findings remain inconsistent, partly due to variations in measurement tools, participant characteristics, and analytical approaches [19]. To address these gaps, this study sought to extend the current body of knowledge by systematically investigating the associations between 24-h MBs and objectively assessed EF subdomains in preschool children. This study employed compositional data analysis and isotemporal substitution modeling to quantify the potential effects of reallocating time among different MBs on EF outcomes. We hypothesized that reallocating time from SB to sleep or MVPA would be associated with more favorable EF scores. The anticipated results may advance theoretical understanding and offer an empirical foundation for developing integrated interventions to promote EF in early childhood.

## 2. Materials and methods

This study is a cross-sectional observational study designed to explore the associations between isotemporal substitution of 24-hour MBs and EF in preschool children. All study procedures, including participant recruitment, informed consent, testing protocols, and safety contingency plans, were reviewed and approved by the Human Research Ethics Committee of East China Normal University (Approval No. HR 342–2024). Prior to data collection, written informed consent was obtained from a parent or guardian of each participating child.

### 2.1. Participants

From September 9, 2024, to January 24, 2025, a total of 371 preschool children meeting the broad eligibility criteria (aged 3–6 years) were initially contacted via convenience sampling from one public and two private kindergartens in Changsha City. The study’s inclusion criteria required children to be enrolled in and expected to remain at the participating kindergartens throughout the data collection period, without major health conditions that could significantly impact PA or cognitive function, and to have written informed consent provided by a parent or legal guardian. To ensure the feasibility and validity of the EF assessments, recruitment was specifically targeted at children in middle and senior classes (approximately 4–6 years old). Children in junior classes (typically around 3–4 years old) were excluded based on pilot testing and related literature [9], which indicated that the assessment battery would be excessively time-consuming and overly difficult for this younger age group, potentially compromising data validity and child compliance. Following this focused recruitment approach, written informed consent was obtained from parents of 319 children.

### 2.2. Methods

#### 2.2.1. 24-h MB Monitoring.

A triaxial accelerometer (ActiGraph GT3X-BT, Pensacola, FL, United States) was used to obtain objective measures of PA and SB. This device provides estimates of light-intensity PA (LPA) and MVPA. Children were asked to wear the accelerometer on the right hip at the level of the iliac crest for seven consecutive days (five weekdays and two weekend days). Before data collection, parents and teachers received detailed instructions, highlighting that the device should be worn continuously, except during sleep or water-based activities.

Raw data were processed using ActiLife software. Non-wear time was determined using the Choi algorithm [20], and data were considered valid if children had at least three days of recording, including two weekdays and one weekend day, with a minimum daily wear time of 480 min [19]. The epoch length was set as 15 s. Activity intensity cutoff points followed the thresholds proposed by Chang & Wang (2021): SB = 0–116 counts/15 s, LPA = 117–551 counts/15 s, Moderate-Intensity PA = 552–997 counts/15 s, and Vigorous-Intensity PA ≥ 998 counts/15 s. These criteria have been validated in Chinese preschoolers aged three to six years [21].

Sleep duration was determined using an online collaborative sleep log jointly completed by the parents, head teachers, and classroom teachers. The log captured the children’s bedtimes, wake-up times, and nap onset and offset. Adapted from Zhenya and Ling (2023), this log displayed strong agreement with ActiGraph wGT3X-BT sleep estimates derived from the Tudor-Locke algorithm (r = 0.972, *p* < 0.001) [22].

#### 2.2.2. EF Assessment.

EF was assessed using the Early Years Toolbox on an iPad. The Early Years Toolbox is a validated and user-friendly tool that supports offline multi-device use and employs a gamified design to enhance children’s engagement [23]. Assessments were conducted in a quiet, well-lit room, with each child accompanied by a trained assessor who provided standardized instructions.

The Early Years Toolbox included three core tasks. (1) Card sorting: This task evaluates cognitive flexibility. Children were required to sort cards based on color or shape, with the sorting rule changing partway throughout the task. The score is based on the number of correct responses following the rule switches. (2) Go/No-Go: This task measures inhibitory control. Children were instructed to respond (“Go”) when a fish appeared and withhold response (“No-Go”) when a shark appeared. The probabilities of the fish and shark stimuli were 80% and 20%, respectively. The inhibitory control score was calculated as the product of the accuracy in Go and No-Go trials. (3) Mr. Ant: This task assesses working memory. Children had to remember the locations of the stickers on an ant’s body and identify them after a delay. The task included eight difficulty levels, with points awarded for each completed level. The task was terminated after three consecutive failures. All three tasks demonstrated good criterion-related validity and were significantly correlated with the NIH Toolbox measures of working memory (r = 0.46), inhibitory control (r = 0.40), and cognitive flexibility (r = 0.45; all *p* < 0.001) [23]. Assessment Duration: A pilot study indicated that completing the three core tasks of the Early Years Toolbox (inhibitory control, working memory, and cognitive flexibility) took approximately 40–45 minutes per child. To minimize the impact of child fatigue or inattention on data quality, all assessments were conducted one-on-one by trained evaluators in a quiet room, and the following standardized measures were implemented: (a) scheduling assessments during the morning hours when children were at their optimal mental state; (b) providing brief rest intervals between each task; and (c) evaluators using standardized encouraging language to maintain children’s engagement. These measures effectively ensured the completeness and reliability of the data [9].

#### 2.2.3. Covariates.

Covariates included sex, age, and body mass index (BMI) of children. Sex and age information were obtained from class rosters. Height and weight were measured by kindergarten staff using standardized equipment (Jianmin brand) one week before testing. BMI was calculated using the following formula: BMI = weight (kg)/height (m^2^). The child’s body mass index was included as a covariate based on existing literature indicating its associations with both MBs [24] and EF [25] development. Controlling for BMI in the statistical analysis aimed to reduce its potential confounding effect on the relationship between the core variables, thereby improving the accuracy of the model estimates.

### 2.3. Data analysis

Data processing and statistical analyses were conducted as follows. First, based on the wearing requirement, theoretical sleep duration was defined as 1440 min minus wear time. Data with a difference exceeding 10% between log-reported and theoretical sleep duration were then excluded, following the method of Weijs et al. [26]. Final sleep duration was determined as follows: if log-reported sleep duration plus wear time was ≥ 1440 min, sleep duration was defined as the theoretical value; otherwise, the log-reported value was adopted, and the remaining time was proportionally allocated to each intensity of PA [27].

Second, descriptive statistics and independent-samples t-tests were employed to examine differences in EF and its subcomponents across demographic variables. Children’s weight status was classified based on the World Health Organization’s definition of overweight for children under five years old [28].

Third, compositional data analysis was applied to describe the composition of 24-h MBs, including measures of central tendency (compositional mean) and dispersion (log-ratio variance). Since compositional data must undergo log-ratio transformation for analysis, and log-ratios require strictly non-zero observations, the zero values in this study were processed using a log-normal mixed model validated by scholars [29].

Fourth, MBs data were transformed using the isometric log-ratio transformation to investigate the associations between different MBs and EF. Regression models were established as follows: E(Y|Z) = β₀ + β₁z₁ + β₂z₂ + β₃z₃ + … + β(d-1)z(d-1) + Covariates; where Y represents EF or its subcomponents, d is the number of MB components, z_i_ are the isometric log ratio transformed variables, and β_i_ are the corresponding regression coefficients. The β values and their 95% confidence intervals are reported as the primary effect sizes in this study. The covariates included sex, age, and BMI.

Finally, R software (version 4.3.1) was used to analyze the isometric substitution effects of reallocating time between different MBs on EF, following the approach proposed by Dumuid et al. [30,31]. Following methodological approaches used in prior studies [9,19], an isotemporal substitution of 15 minutes per day was applied to analyze the relationship between the reallocation of time among different MBs and EF.

## 3. Results

### 3.1. Sample characteristics

Of the initial cohort, 266 participants provided valid data across all 24-hour MBs assessment and EF evaluations, constituting the final analytical sample. Attrition occurred primarily at two stages: 52 children were excluded due to parental lack of time or interest during the consent process, and a further 53 children were excluded during data collection—31 for incomplete device wear or logs, and 22 for insufficient data validity (e.g., inadequate accelerometer wear days).

The demographic characteristics of the final sample are summarized in Table 1. In brief, the sample was balanced in sex (approximately 50% boys and 50% girls) and age. Slightly more children attended middle classes than senior classes. The prevalence of overweight exceeded the national average for children under six years reported in the Report on Nutrition and Chronic Disease Status of Chinese Residents (2020) (6.8%). Nearly all children were primarily cared for by parents, with grandparents or other caregivers representing a small proportion (Table 1).

**Table 1:** Demographic characteristics of the participants.

Group	Subgroup	N	%/Mean ± SD
Sex	Boy	134	50.4
	Girl	132	49.6
Age	3-4	134	50.4
	5-6	132	49.6
	Total (Mean ± SD)	266	4.56 ± 0.66
Class	Middle class	142	53.4
	Senior class	124	46.6
BMI status	Overweight	28	10.5
	Non-overweight	238	89.5
Primary Caregiver	Parent(s)	260	97.7
	Grandparent(s)	5	1.9
	Other	1	0.4

### 3.2. Basic characteristics of EF in preschool children

Girls exhibited significantly higher inhibitory control than boys (t = –3.43, *p* = 0.001), with no significant differences in cognitive flexibility (t = 0.78, *p* = 0.434) or working memory (t = –0.85, *p* = 0.395).

Regarding age, preschool children aged three–four years performed significantly lower than those aged five–six years across all three EF dimensions, inhibitory control (t = –2.43, *p* = 0.016), cognitive flexibility (t = –3.05, *p* = 0.003), and working memory (t = –3.19, *p* = 0.002), indicating that EF improves with age.

No significant differences were observed in overall EF or any of its subcomponents between overweight and non-overweight children (all p > 0.05) (Table 2).

**Table 2:** EF status of preschool children.

Group	Subgroup	Inhibitory control	Cognitive flexibility	Working memory
Sex	Boy	0.85 ± 0.13	10.39 ± 1.40	2.48 ± 0.76
	Girl	0.89 ± 0.87	10.24 ± 1.76	2.56 ± 0.70
	Mean difference (95% CI)	−0.05 (−0.07, −0.02)	0.15 (−0.23, 0.54)	−0.08 (−0.25, 0.10)
	p	0.001	0.434	0.395
Age	3–4	0.85 ± 0.13	10.03 ± 1.76	2.38 ± 0.78
	5–6	0.89 ± 0.09	10.61 ± 1.33	2.66 ± 0.65
	Mean difference (95% CI)	−0.03 (−0.06, −0.01)	−0.58 (−0.96, −0.21)	−0.28 (−0.45, −0.11)
	p	0.015	0.003	0.002
BMI	Overweight	0.86 ± 0.08	10.71 ± 1.12	2.43 ± 0.80
	Non-overweight	0.87 ± 0.12	10.27 ± 1.63	2.53 ± 0.72
	Mean difference (95% CI)	−0.01 (−0.06, 0.03)	0.44 (−0.18, 1.06)	−0.10 (−0.39, 0.19)
	p	0.60	0.16	0.48
Total		0.87 ± 0.11	10.32 ± 1.58	2.52 ± 0.73

Note: Data are presented as mean ± standard deviation (M ± SD). Group comparisons were performed using independent samples t-tests. No post-hoc analyses were conducted, and no adjustments for multiple comparisons (e.g., Bonferroni correction) were applied, as these analyses were exploratory. BMI classification (overweight vs. Non-overweight) followed WHO standards. Abbreviations: CI, confidence interval

### 3.2. Characteristics of 24-h MBs in preschool children

The average composition of 24-h MBs among preschool children is presented in Table 3. Sleep accounted for 42.11% of the day, SB for 44.10%, LPA for 10.84%, and MVPA for 2.94% (Table 3).

**Table 3:** Compositional descriptive statistics of 24-h MBs: central tendency and dispersion measures.

Central tendency	Sleep	SB	LPA	MVPA
Composition Mean (min/day)	606.44	635.04	156.15	42.36
Composition/%	42.11	44.10	10.84	2.94
Variation matrix				
Sleep	0.00	0.08	0.05	0.22
SB	0.08	0.00	0.14	0.38
LPA	0.05	0.14	0.00	0.14
MVPA	0.22	0.38	0.14	0.00

Notes: The first two rows present the compositional central tendency (compositional means). The lower 4 × 4 matrix (rows Sleep to MVPA) is the variation matrix, showing pairwise log-ratio variances between each pair of movement behaviors. In compositional data analysis, the variation matrix – defined as the log-ratio variance between all pairwise components – is used to describe the dispersion of the data. A smaller variance indicates that the time allocation ratio between the corresponding two behaviors is more unstable, with a higher probability of mutual substitution; a larger variance suggests that the ratio is more stable, with a lower probability of mutual substitution. The log-ratio variance is unitless and has no physical unit of measurement. Abbreviations: SB, sedentary behavior; LPA, light intensity physical activity; MVPA, moderate-to-vigorous physical activity.

The variation matrix analysis indicated that the log-ratio variance between SB and MVPA was the highest (0.38), suggesting a relatively high variability and low dependency between these two behaviors. Changes in MVPA time were more likely to be compensated by changes in LPA (log-ratio variance = 0.14). However, alterations in sleep time were likely replaced by SB (log-ratio variance = 0.08) and LPA (log-ratio variance = 0.05).

### 3.3. Associations Between 24-h MBs and EF

The associations between 24-h MBs and EF are presented in Table 4. The relative composition of 24-h MBs was significantly associated with inhibitory control, cognitive flexibility, and working memory (all *p* < 0.001), with model R² values > 10% for each EF dimension. The highest model explanatory power was observed for inhibitory control (R² = 16.3%). After controlling for other MBs, SB was negatively associated with inhibitory control [γ_1_^2^ = –0.11 (−0.17, −0.05), *p* < 0.001], cognitive flexibility [γ_1_^2^ = –1.45 (−2.29, −0.61), *p* = 0.001], and working memory [γ_1_^2^ = –0.58 (−0.97, −0.19), *p* = 0.004]. In contrast, sleep was positively associated with cognitive flexibility [γ_1_^1^ = 1.60 (0.26, 2.94), *p* = 0.020; Table 4]

**Table 4:** Associations between 24-h MBs and EF.

	Sleep		SB		LPA		MVPA		Model	Adjust R^2^
	γ_1_^1^	*P*	γ_1_^2^	*P*	*γ*_*1*_^*3*^	*P*	γ_1_^4^	*P*	*P*	
Inhibitory control	0.05 (−0.04,0.14)	0.285	–0.11 (−0.17,-0.05)	< 0.001	0.04 (−0.04,0.12)	0.312	0.02 (−0.02,0.06)	0.385	< 0.001	16.3
Cognitive flexibility	1.60 (0.26,2.94)	0.020	–1.45 (−2.29,-0.61)	0.001	0.14 (−1.02,1.30)	0.817	–0.28 (−0.88,0.31)	0.351	< 0.001	10.7
Working memory	0.33 (−0.29,0.95)	0.293	–0.58 (−0.97,-0.19)	0.004	0.04 (−0.49,0.58)	0.875	0.20 (−0.07,0.48)	0.150	< 0.001	10.1

Notes: Values are regression coefficients (γ) with 95% confidence intervals shown in parentheses, obtained from compositional data analysis using isometric log-ratio (ilr) transformation. The γ coefficients represent the associations between each 24-hour movement behavior (Sleep, SB, LPA, MVPA) and executive function outcomes. All models were adjusted for covariates including sex, age, and BMI. Model P values indicate the overall significance of the regression model. Adjusted R² values represent the proportion of variance explained by the model. Abbreviations: SB, sedentary behavior; LPA, light intensity physical activity; MVPA, moderate-to-vigorous physical activity; EF, executive function.

### 3.4. Changes in EF Following 15-min/day Isotemporal Substitution of 24-h MBs

It is worth noting that among the various isotemporal substitution pathways examined, only a few reached statistical significance, although some significant findings were also observed. The results indicated that replacing SB with sleep 15 min/day increased the EF scores of preschool children: inhibitory control increased by 0.003 (0.0004, 0.006), and cognitive flexibility increased by 0.064 (0.021, 0.106). Conversely, reallocating time in the opposite direction resulted in significant decreases in the EF scores (Table 5).

**Table 5:** Effects of 15-min daily time reallocation between 24-h MBs on EF in preschool children.

+15 min	–15 min	Inhibitory control	Cognitive flexibility	Working memory
Sleep	SB	0.003* (0.0004,0.006)	0.064* (0.021,0.106)	0.019 (−0.0007,0.039)
Sleep	LPA	–0.003 (−0.011,0.006)	0.022 (−0.098,0.142)	0.003 (−0.052,0.059)
Sleep	MVPA	–0.006 (−0.022,0.010)	0.141 (−0.091,0.373)	–0.070 (−0.177,0.038)
SB	LPA	–0.006 (−0.013,0.001)	–0.041 (−0.145,0.063)	–0.015 (−0.064,0.033)
SB	MVPA	–0.009 (−0.024,0.006)	0.078 (−0.143,0.299)	–0.088 (−0.190,0.014)
SB	Sleep	–0.003* (−0.006,-0.0003)	–0.064* (−0.107,-0.021)	–0.019 (−0.039,0.001)
LPA	SB	0.005 (−0.001,0.012)	0.041 (−0.054,0.136)	0.015 (−0.029,0.059)
LPA	MVPA	–0.004 (−0.024,0.016)	0.118 (−0.171,0.407)	–0.073 (−0.207,0.061)
LPA	Sleep	0.002 (−0.005,0.010)	–0.024 (−0.135,0.087)	–0.004 (−0.055,0.048)
MVPA	SB	0.007 (−0.003,0.018)	–0.044 (−0.196,0.108)	0.065 (−0.005,0.135)
MVPA	LPA	0.001 (−0.015,0.017)	–0.086 (−0.317,0.144)	0.049 (−0.057,0.156)
MVPA	Sleep	0.004 (−0.008,0.015)	–0.109 (−0.273,0.055)	0.046 (−0.030,0.122)

Notes: This table presents the results of isotemporal substitution analysis following the method proposed by Dumuid et al. The values represent the estimated change in executive function (EF) scores (with 95% confidence intervals shown in parentheses) when 15 minutes per day is reallocated from the behavior listed in the “–15 min” column to the behavior listed in the “+15 min” column. Positive values indicate that reallocating time to the “+15 min” behavior (and away from the “–15 min” behavior) is associated with higher EF scores; negative values indicate an association with lower EF scores. * p < 0.05 (statistically significant). Abbreviations: SB, sedentary behavior; LPA, light physical activity; MVPA, moderate-to-vigorous physical activity; EF, executive function.

## 4. Discussion

This study employed compositional data analysis to systematically investigate the relationship between the overall composition of 24-hour MBs and objectively assessed EF in preschool children. The main findings include: first, the overall pattern of 24-hour time composition was significantly associated with all three core subcomponents of EF (inhibitory control, cognitive flexibility, and working memory), highlighting the importance of analyzing these behaviors as an integrated system; second, isotemporal substitution model analysis revealed that reallocating 15 minutes per day from SB to sleep was significantly associated with improvements in inhibitory control and cognitive flexibility scores. This finding provides methodological support for the “time reallocation” hypothesis and offers specific quantitative guidance for behavioral interventions. Notably, the majority of time reallocation pathways in the model did not reach statistical significance (Table 5), which may suggest that EF in generally healthy preschool children is relatively insensitive to minor adjustments in daily activity time, or that detecting such effects requires a larger sample size or longer-term observation.

Specifically regarding various types of behaviors, this study found that sleep and SB are key behaviors closely linked to EF in preschool children. This aligns with existing evidence: prior systematic reviews have reported a modest positive correlation between sleep and EF [32], a finding corroborated by our results which indicate that, after controlling for other behaviors, sleep remained positively associated with cognitive flexibility. Notably, substituting 15 minutes per day of sedentary time with sleep was associated with significant improvements in both inhibitory control and cognitive flexibility, thereby reinforcing the potential benefits of this specific behavioral reallocation.

The effects of SB warrant further investigation. The existing literature presents some inconsistencies on this issue. While a review of 16 studies indicated that seven reported negative associations between SB and EF and two reported positive associations [33], the findings of the current study are clear and align with the predominant trend in the literature: SB demonstrated a significant negative association with all three dimensions of EF. This consistency underscores the potential detrimental role of excessive sedentary time in early cognitive development. From a neurobiological perspective, SB may impair white matter development, which is crucial for EF [34]. Given that myelination and neural signal conduction efficiency are influenced by environmental factors [35], prolonged SB may negatively impact EF development.

However, we must interpret the associations of SB with caution. Different types of sedentary activities may show divergent associations with EF; engagement in cognitively stimulating activities (for instance, puzzles or problem-solving games) has been associated with better EF [36], whereas time spent in passive screen-based activities has been associated with poorer outcomes [33]. Neuroimaging studies have demonstrated that excessive screen time is associated with reduced frontal gray matter volume and decreased white matter anisotropy [34,37], and the frontal lobe is a key brain region for cognitive flexibility [13]. Overall, research on the neural mechanisms linking SB, especially cognitive flexibility and EF, remains limited and warrants further investigation [13,33].

Unlike SB and sleep, the relationship between PA and EF presents a more complex picture. Unlike the strong “PA-EF” associations observed in some studies [9,10], the present study found relatively limited contributions from PA variables, a discrepancy that may be related to differences in sample characteristics or measurement contexts. The current empirical evidence regarding the association between PA and EF has not yet reached a consensus. For example, some studies have shown a significant positive correlation between moderate-to-vigorous PA and inhibitory control [9], while other research has found a significant positive correlation between vigorous intensity PA and cognitive flexibility [15]. Still other studies have reported no significant association between PA and inhibitory control or cognitive flexibility, only a negative correlation with working memory, and have emphasized that such associations may be limited by specific environments and types of activities [14]. Notably, the heterogeneity in findings may also stem from methodological diversity. For instance, a study employing multivariate pattern analysis revealed highly complex nonlinear relationships between PA intensity and EF [38], some of which were even difficult to explain directly using existing theories. In contrast, compositional data analysis offers a novel and complementary research perspective for understanding these complex associations by focusing on the relative proportions and structural balance of time allocation.

The value of this study is primarily reflected in two aspects. At the measurement level, compared to our team’s previous reliance on parent-reported behavioral assessments [19], this study utilized standardized cognitive tasks administered via tablets for direct measurement. This approach avoids the subjective bias inherent in third-party reports and provides a more precise reflection of real-time performance in core cognitive components. At the methodological level, the adoption of the compositional data analysis framework addresses the multicollinearity issues among behavioral variables commonly encountered in traditional regression analysis [39]. This allows for more rigorous statistical support for composite health recommendations such as “reducing SB and increasing sleep.”

This study has several limitations. First, there is a limitation in the study design. This study employed a cross-sectional design, which precludes causal inference between variables. Although we hypothesized that MBs may influence the development of EF, the reverse explanation—that children with better EF may tend to choose healthier activity patterns—is equally plausible. Second, there are limitations in the measurement methods. The measurement of sleep duration relied primarily on parent-reported logs. Although preliminary validation showed a high correlation with device-monitored data [22], recall bias cannot be completely ruled out. Third, there is a limitation in sample representativeness. The study sample was recruited through convenience sampling from kindergartens in eastern Chinese cities. The generalizability of the findings to rural areas, children from different cultural backgrounds, or varying socioeconomic backgrounds requires further verification. Fourth, there was inadequate control for covariates. Due to parental concerns about privacy, key socioeconomic indicators such as household monthly income had a high missing data rate (up to 19.6%), which may have limited our ability to control for potential confounding effects of socioeconomic status..

## 5. Conclusions

The present study demonstrates that the 24-h MBs composition significantly predicts EF in preschool children, with sleep showing a positive association and SB a negative association. Reallocating time from SB to sleep is associated with improved cognitive flexibility. These findings have important practical implications: given that preschool children’s daily movement patterns are highly modifiable, interventions targeting sleep enhancement may yield tangible cognitive benefits. Notably, the lack of significant effects for most other substitution pathways suggests that sleep may play a unique and non-substitutable role in preschool EF. Future research should employ longitudinal and experimental designs to establish causality, investigate the neurobiological mechanisms linking sleep to EF in young children, and examine whether modest increases in sleep duration produce measurable cognitive improvements.

## Supporting information

S1 File Article Data. This is the supporting data for the manuscript.
(XLS)

## Abbreviations

MBs: Movement Behaviors
PA: Physical Activity
LPA: Light-Intensity Physical Activity
MVPA: Moderate-to-Vigorous Physical Activity
SB: Sedentary Behavior
EF: Executive Function
BMI: Body Mass Index

## References

[1] Posne MI, Rothbart MK. Developing mechanisms of self-regulation. Dev Psychopathol. 2000;12(3):427–41. doi: 10.1017/s0954579400003096 11014746

[2] Li M, Bai X. Research progress on the development of cognitive flexibility in executive function. Psychological Exploration. 2005;25(2):35–8.

[3] Miyake A, Friedman NP, Emerson MJ, Witzki AH, Howerter A, Wager TD. The unity and diversity of executive functions and their contributions to complex “Frontal Lobe” tasks: a latent variable analysis. Cogn Psychol. 2000;41(1):49–100. doi: 10.1006/cogp.1999.0734 10945922

[4] Diamond A. Why assessing and improving executive functions early in life is critical: Executive function in preschool-age children: Integrating measurement, neurodevelopment and translational research. 2015.

[5] Graziano PA, Garb LR, Ros R, Hart K, Garcia A. Executive functioning and school readiness among preschoolers with externalizing problems: The moderating role of the student–teacher relationship. Early Education and Development. 2015;27(5):573–89. doi: 10.1080/10409289.2016.1102019

[6] Nelson TD, Nelson JM, James TD, Clark CAC, Kidwell KM, Espy KA. Executive control goes to school: Implications of preschool executive performance for observed elementary classroom learning engagement. Dev Psychol. 2017;53(5):836–44. doi: 10.1037/dev0000296 28358540 PMC5436807

[7] Moffitt TE, Arseneault L, Belsky D, Dickson N, Hancox RJ, Harrington H, et al. A gradient of childhood self-control predicts health, wealth, and public safety. Proc Natl Acad Sci U S A. 2011;108(7):2693–8. doi: 10.1073/pnas.1010076108 21262822 PMC3041102

[8] Diamond A, Ling DS. Conclusions about interventions, programs, and approaches for improving executive functions that appear justified and those that, despite much hype, do not. Dev Cogn Neurosci. 2016;18:34–48. doi: 10.1016/j.dcn.2015.11.005 26749076 PMC5108631

[9] Bezerra TA, Clark CCT, Souza Filho AND, Fortes LDS, Mota JAPS, Duncan MJ, et al. 24-hour movement behaviour and executive function in preschoolers: A compositional and isotemporal reallocation analysis. Eur J Sport Sci. 2021;21(7):1064–72. doi: 10.1080/17461391.2020.1795274 32654601

[10] McNeill J, Howard SJ, Vella SA, Cliff DP. Compliance with the 24-Hour movement guidelines for the early years: Cross-sectional and longitudinal associations with executive function and psychosocial health in preschool children. J Sci Med Sport. 2020;23(9):846–53. doi: 10.1016/j.jsams.2020.02.011 32360244

[11] Kuzik N, Poitras VJ, Tremblay MS, Lee EY, Hunter S, Carson V. Systematic review of the relationships between combinations of movement behaviours and health indicators in the early years (0-4 years). BMC Public Health. 2017;17(Suppl 5):849. 29219071 10.1186/s12889-017-4851-1 PMC5773877

[12] Bernier A, Cimon-Paquet C, Tétreault É. Sleep development in preschool predicts executive functioning in early elementary school. Adv Child Dev Behav. 2021;60:159–78. doi: 10.1016/bs.acdb.2020.08.005 33641792

[13] Cui J, Li L, Dong C. The associations between specific-type sedentary behaviors and cognitive flexibility in adolescents. Front Hum Neurosci. 2022;16:910624. doi: 10.3389/fnhum.2022.910624 36034120 PMC9411862

[14] Cook CJ, Howard SJ, Scerif G, Twine R, Kahn K, Norris SA, et al. Associations of physical activity and gross motor skills with executive function in preschool children from low-income South African settings. Dev Sci. 2019;22(5):e12820. doi: 10.1111/desc.12820 30801916

[15] McNeill J, Howard SJ, Vella SA, Cliff DP. Longitudinal associations of physical activity and modified organized sport participation with executive function and psychosocial health in preschoolers. J Sports Sci. 2020;38(24):2858–65. doi: 10.1080/02640414.2020.1803037 32912077

[16] Kuzik N, Spence JC, Arkko K, Blye C-J, Davie J, Duddridge R, et al. Associations between meeting the Canadian 24-hour movement guidelines and physical, cognitive, social-emotional, and overall development in early childhood. J Act Sedentary Sleep Behav. 2022;1(1):2. doi: 10.1186/s44167-022-00002-4 40229982 PMC11934447

[17] Lau PWC, Song H, Song D, Wang J-J, Zhen S, Shi L, et al. 24-Hour movement behaviors and executive functions in preschoolers: A compositional and isotemporal reallocation analysis. Child Dev. 2024;95(2):e110–21. doi: 10.1111/cdev.14013 37787120

[18] Lu Z, Qu X, Chang J, Xu M, Song G, Wang X, et al. Reallocation of time between preschoolers’ 24-h movement behaviours and executive functions: A compositional data analysis. J Sports Sci. 2023;41(12):1187–95. doi: 10.1080/02640414.2023.2260632 37724814

[19] Chang Z, Wang L, Zhu A. Relationship between 24-hour movement behaviors and executive function in preschool children based on compositional data analysis. Acta Psychol (Amst). 2025;259:105394. doi: 10.1016/j.actpsy.2025.105394 40812261

[20] Choi L, Liu Z, Matthews CE, Buchowski MS. Validation of accelerometer wear and nonwear time classification algorithm. Med Sci Sports Exerc. 2011;43(2):357–64. doi: 10.1249/MSS.0b013e3181ed61a3 20581716 PMC3184184

[21] Chang Z, Wang S. Calibration, validation, and application of the optimal cut-off points for activity counts in diagnosing physical activity intensity in preschool children. Journal of Capital University of Physical Education and Sports. 2021;33(1):74–83.

[22] Zhenya C, Ling W. A comparative study of different sleep assessment methods for preschool children. Am J Hum Biol. 2023;35(10):e23936. doi: 10.1002/ajhb.23936 37335269

[23] Howard SJ, Melhuish E. An early years toolbox for assessing early executive function, language, self-regulation, and social development: validity, reliability, and preliminary norms. J Psychoeduc Assess. 2017;35(3):255–75. 28503022 10.1177/0734282916633009 PMC5424850

[24] Taylor RW, Haszard JJ, Meredith-Jones KA, Galland BC, Heath A-LM, Lawrence J, et al. 24-h movement behaviors from infancy to preschool: Cross-sectional and longitudinal relationships with body composition and bone health. Int J Behav Nutr Phys Act. 2018;15(1):118. doi: 10.1186/s12966-018-0753-6 30477518 PMC6260686

[25] Zhou Y, Li R, Zha J, Wu J, Wan Y, Huang Y. Associations of body mass index and screen exposure with executive function in preschool children. Chinese Journal of School Health. 2024;45(8):1111–4, 9.

[26] Weijs PJM. Validity of predictive equations for resting energy expenditure in US and Dutch overweight and obese class I and II adults aged 18-65 y. Am J Clin Nutr. 2008;88(4):959–70. doi: 10.1093/ajcn/88.4.959 18842782

[27] Huang B, Tan J, Liu Q, Zhang D, Xu H, Huang Z, et al. Comparison and empirical study of the application of compositional isotemporal substitution model and non-compositional isotemporal substitution model in the research field of health effects of physical activity. China Sport Science. 2022;42(2):51–8.

[28] WHO. Definition of overweight and obesity 2025.https://www.who.int/zh/news-room/fact-sheets/detail/obesity-and-overweight

[29] Bear J, Billheimer D. A Logistic normal mixture model for compositional data allowing essential zeros. AJS. 2016;45(4):3–23. doi: 10.17713/ajs.v45i4.117

[30] Dumuid D, Stanford TE, Pedišić Ž, Maher C, Lewis LK, Martín-Fernández J-A, et al. Adiposity and the isotemporal substitution of physical activity, sedentary time and sleep among school-aged children: A compositional data analysis approach. BMC Public Health. 2018;18(1):311. doi: 10.1186/s12889-018-5207-1 29499689 PMC5834855

[31] Dumuid D, Pedišić Ž, Stanford TE, Martín-Fernández J-A, Hron K, Maher CA, et al. The compositional isotemporal substitution model: A method for estimating changes in a health outcome for reallocation of time between sleep, physical activity and sedentary behaviour. Stat Methods Med Res. 2019;28(3):846–57. doi: 10.1177/0962280217737805 29157152

[32] Reynaud E, Vecchierini M-F, Heude B, Charles M-A, Plancoulaine S. Sleep and its relation to cognition and behaviour in preschool-aged children of the general population: A systematic review. J Sleep Res. 2018;27(3):e12636. doi: 10.1111/jsr.12636 29164715

[33] Li S, Guo J, Zheng K, Shi M, Huang T. Is sedentary behavior associated with executive function in children and adolescents? A systematic review. Front Public Health. 2022;10:832845. doi: 10.3389/fpubh.2022.832845 35186852 PMC8847290

[34] Hutton JS, Dudley J, Horowitz-Kraus T, DeWitt T, Holland SK. Associations between screen-based media use and brain white matter integrity in preschool-aged children. JAMA Pediatr. 2020;174(1):e193869. doi: 10.1001/jamapediatrics.2019.3869 31682712 PMC6830442

[35] Forbes TA, Gallo V. All wrapped up: Environmental effects on myelination. Trends Neurosci. 2017;40(9):572–87. doi: 10.1016/j.tins.2017.06.009 28844283 PMC5671205

[36] Carson V, Kuzik N, Hunter S, Wiebe SA, Spence JC, Friedman A, et al. Systematic review of sedentary behavior and cognitive development in early childhood. Prev Med. 2015;78:115–22. doi: 10.1016/j.ypmed.2015.07.016 26212631

[37] Zavala-Crichton JP, Esteban-Cornejo I, Solis-Urra P, Mora-Gonzalez J, Cadenas-Sanchez C, Rodriguez-Ayllon M, et al. Association of sedentary behavior with brain structure and intelligence in children with overweight or obesity: The activebrains project. J Clin Med. 2020;9(4):1101. doi: 10.3390/jcm9041101 32290576 PMC7230478

[38] Vabø KB, Aadland KN, Howard SJ, Aadland E. The multivariate physical activity signatures associated with self-regulation, executive function, and early academic learning in 3-5-year-old children. Front Psychol. 2022;13:842271. doi: 10.3389/fpsyg.2022.842271 35478740 PMC9037291

[39] Yang X, Wang L, Xu Q, Chen H, Liang G, Qiu Y. Research on 24-hour activity recommendations for children and adolescents in China based on physical health, mental health, and executive function. Journal of Sports Science. 2024;44(7):75–86.

